# Acute Phase Serum Leptin, Adiponectin, Interleukin-6, and Visfatin Are Altered in Chinese Children With Febrile Seizures: A Cross-Sectional Study

**DOI:** 10.3389/fendo.2020.00531

**Published:** 2020-09-15

**Authors:** Jie-ru Chen, Mei-fang Jin, Ling Tang, Yue-ying Liu, Hong Ni

**Affiliations:** ^1^Division of Brain Science, Institute of Pediatric Research, Children's Hospital of Soochow University, Suzhou, China; ^2^Wuxi Second People's Hospital, Wuxi, China

**Keywords:** febrile seizures, adiponectin, IL-6, leptin, visfatin

## Abstract

Adipokines, including leptin, visfatin, adiponectin, and interleukin-6 (IL)-6, play multiple roles in the pathophysiology of epilepsy and febrile seizures (FS). We aimed to investigate the associations among plasma adipokines, mainly leptin, visfatin, adiponectin, or IL-6, and the prognosis of FS. This prospective cross-sectional study was conducted from January 2017 to December 2018 at the Wuxi Second People' Hospital China. The levels of serum leptin, visfatin, adiponectin, and IL-6 in 55 children with FS (FS group) were compared with 42 febrile children without seizure (FC group) and 48 healthy children (HC group) in an acute phase. The correlation with clinical indicators was determined by logistic regression analysis. Serum adiponectin and IL-6 levels were significantly higher in the FS group than in the FC and HC groups (*p* < 0.05), but there was no statistical difference between the FC and HC groups. In addition, logistic regression analysis showed that high concentrations of adiponectin and IL-6 were significantly associated with the occurrence of FS. For leptin and visfatin, they were significantly lower in the FS and FC groups than in the normal control group, but there was no statistical difference between the FS and FC groups. Our results suggest that higher plasma levels of IL-6 and adiponectin may serve as an additional biomarker in the early treatment or follow-up of the FS children.

## Introduction

Febrile seizures (FS) are one of the most common clinical diseases in pediatric neurology. It occurs between 6 months and 6 years of age and occurs in ~2–5% of children in the United States and Western Europe, ~5–14% in Asian countries, and 3–5% in China (1–3). According to the age, frequency, duration, and type of seizures, FS is divided into simple febrile seizures (SFS) and complex febrile seizures (CFS). SFS accounts for 70–75% of seizures, while CFS accounts for 9–35%. Approximately 30–50% of cases may reoccur after the first attack, and the risk of FS turning into epilepsy is 4–5 times that of the general population (4). Repeated FS can lead to brain injuries, such as motor dysfunction, language disability, behavioral cognitive impairment, and higher risk of epilepsy (4, 5). Therefore, early diagnosis of children with FS is of great significance for early intervention and reduction of neurological sequelae and is an urgent task for pediatric clinics.

With the widespread application of technologies, such as molecular biology, in medicine, some biomarkers for predicting or diagnosing FS have attracted attention. Imuekemhe et al. in 1989 and 1996 found that lactic acid in the serum and cerebrospinal fluid of children with FS was significantly increased (6). Wellmann et al. found that the serum copeptin and Von Willebrand factor (VWF) of children with FS were significantly higher than those of the control group (3, 7). The levels of proinflammatory cytokines, such as interleukin-6 (IL-6) and tumor necrosis factor-α (TNF-α), and anti-inflammatory cytokines, such as interleukin-4 (IL-4), in the peripheral blood of children with FS also exhibited significant changes, suggesting that these inflammatory molecules may play an important role in the pathogenesis of FS (8–10). These observations suggest that serum levels of some bioactive substances have a specific relation with FS, but so far, none has been clinically recognized and applied. Thus, further research is recommended.

At present, research on the role of adipokines in the pathogenesis of FS is receiving attention. Multiple studies have examined changes in serum adipokine levels in children with FS (11–13). However, they have variable results, and there are issues with the studies, such as small numbers of cases, young age, and lack of correlation analysis with clinical indicators. In particular, as far as we know, there are few studies in China to assess the changes of peripheral blood adipokines in children with FS; this may be related to the low education level and low income of parents of children with FS in many areas, which leads to parents' unwillingness to cooperate with the investigation. The present study was completed in Wuxi, in the Jiangnan region, the most developed industrial city in China. Citizens in this area have higher education levels and income, and generally have medical insurance as a guarantee, and the nutritional status of their children is better than that of children in other regions. These combined factors enabled the children's parents to undertake the financial expenses of the investigation, thus ensuring the successful completion of the study.

The purpose of the study is: (1) to look at changes in serum biomarkers and (2) to provide useful data on additional biomarkers during early treatment or follow-up of the FS children.

## Materials and Methods

This was a prospective cross-sectional study performed at the Wuxi Second People' Hospital China from January 2017 to December 2018. Fifty-five children with an FS diagnosed in the same hospital were enrolled in the study. The age of the patients ranged from 9 months to 8 years (mean 34 months). Diagnosis of FS followed the criteria established by American Academy of Pediatrics in 2011 (14). The electroencephalogram (EEG) was normal for all patients or showed mild non-specific abnormalities. FS were divided into two groups, namely, the simple FS (SFS) group (*n* = 42), defined as those with primary generalized seizures that lasted for <15 min and did not recur within 24 h, and the complex FS (CFS) group (*n* = 13), defined as those with seizures that were focal, prolonged (≥15 min), and/or recurrent within 24 h (14). The diseases in the FS group include acute upper respiratory tract infection, acute bronchitis, herpes angina, and acute laryngitis. The study protocol was approved by the Human Ethics Committee of the hospital, and informed consent was obtained from the parents of children.

Children with epilepsy, hereditary metabolic disease, congenital malformation, intracranial space-occupying lesions, intracranial infection, intellectual disability, and other brain injuries were excluded. Patients with diseases known to affect adipocytokines, such as diabetes, genetic syndromes (such as glycogen accumulation disease, mucopolysaccharidosis, Gaucher disease, and other diseases of sugar or lipid metabolism disorders), obese patients, and gastrointestinal diseases, such as diarrhea, were also excluded.

Ninety healthy children of comparable age and sex, without a history of febrile or afebrile seizures, were enrolled as a control group. Children with original heart, brain, endocrine, and other basic diseases were excluded. We subdivided our control children into two groups.

The FC group (*n* = 42): children hospitalized at our pediatric department with fever due to infection, except for central nervous system infection. Diseases in the FC group included acute upper respiratory tract infections, acute bronchitis, acute laryngitis, and bronchopneumonia. Because the children in the FS group were all suffering from respiratory diseases, the children in the FC group were also selected children with respiratory diseases, excluding children with gastrointestinal diseases, such as diarrhea. Children with otitis media, diarrhea, skin rash, electrolyte disturbance, and other complications were also excluded.

In addition, hyponatremia-induced convulsions mostly occur in moderate (125–130 mmol/L) to severely low sodium levels (<125 mmol/L), so this study excluded children with serum sodium <130 mmol/L in both the FS and FC groups.

HC group (*n* = 48): children who attended pediatric outpatient clinics for routine physical examinations with no history of illness for 1 month before and after the physical examination.

All patients and controls had appropriate medical history and received comprehensive clinical and detailed neurological examinations. Laboratory studies were performed on all children, including complete blood count (CBC), C-reactive protein (CRP), and body temperature (in Celsius) at admission.

Venous blood samples (5 ml, non-fasting status) were obtained from the patient within 3 h after the seizure. Two-milliliter samples were collected for CBC, CRP, glucose, and sodium detection. Electrolytes such as potassium, calcium, magnesium, and chlorine were also tested. Because the cases of electrolyte disturbance were excluded, the blood electrolytes of the enrolled cases were within the normal range, so no statistical analysis was done. The remaining blood samples were centrifuged, and the serum was stored in EP tubes below −80°C without anticoagulation treatment until use. Control samples were collected immediately from the vein and similarly stored and analyzed.

Serum levels of leptin were assessed using the solid phase sandwich enzyme-linked immunosorbent assay (ELISA) according to the manufacturer's instructions (ELISA kit, Invitrogen, California, USA). The intra-assay coefficient of variation (CV) value of leptin was 3.9%, and the inter-assay coefficient of variation was 5.3%.

Serum adiponectin levels were measured using ELISA technology (eBioscience, California, USA). The intra-assay CV value was 4.2% and the inter-assay CV was 3.1% for adiponectin.

The serum levels of visfatin were detected with a commercially available ELISA kit (LifeSpan Biosciences, Seattle, USA). The intra-assay CV value was <10%, and the inter-assay CV was <15% for visfatin.

The serum IL-6 concentration was assessed according to the manufacturer's instructions by quantitative sandwich enzyme immunoassay technology (ELISA kit provided by Multi sciences, Hangzhou, China). The intra-assay CV value was 5.0% and the inter-assay CV was 4.6% for IL-6.

### Statistical Analysis

The statistical evaluation was conducted using the SPSS software version 17.0 (SPSS Inc. Chicago, Illinois, USA). Categorical variables are shown as frequencies and compared with the chi-squared test, and continuous data were expressed as mean ± standard deviation (normal distribution) or median (min–max) (non-normal distribution) to better meet the statistics principle. Test selection was based on evaluating the variables for normal distribution using the Kolmogorov–Smirnov test. An independent *t*-test and Mann-Whitney *U*-test were used to analyze data between two groups. One-way analysis of variance (ANOVA) test followed by the least significant difference (LSD) and Kruskal-Wallis test followed by Nemenyi test were carried out to compare more than two independent groups (FS, FC, and HC). Spearman's correlation was used to test the correlation analysis. Binomial logistic regression analysis was used to define the association between febrile seizures (as the dependent variable) and estimated plasma adipocytokines levels (as the independent variables). Statistical significance was set at *p* < 0.05.

## Results

Table 1 shows the comparison of selected clinical and laboratory data between the FS group, the FC group, and the control group. The study included 55 FS patients (mean age 2.9 ± 1.8 years)−13 of whom had complex FS (CFS) (mean age 3.6 ± 2.3)−42 FC patients (mean age 3.5 ± 1.8), and 48 healthy children who served as controls (HC, mean age 3.4 ± 2.0). The FS, FC, and HC groups were found to be comparable with regard to the age, the ratios of sex, white blood cell (WBC) count, neutrophil ratio, CRP, and serum glucose (*p* > 0.05). However, hemoglobin, the platelet count and serum sodium level were found to be significantly lower in the FS group than in the FC and HC groups (*p* < 0.05).

**Table 1:** Comparison of clinical and laboratory findings of patient and control group^e^.

	**FS group (*n* = 55)**	**Fc group (*n* = 42)**	**Hc group (*n* = 48)**	***P-*value**
Age (months)	35.38 ± 21.19	42.52 ± 21.40	41.35 ± 24.50	>0.05^*^
Sex (male/female)^d^	32/23	22/20	26/22	>0.05
BT (°C) on admission	39.39 ± 0.65^b^	39.34 ± 0.57^c^	36.69 ± 0.27^a^	<0.05^*^
WBC (×10^9^/L)	6.00 (2.70–21.65)	7.94 (2.10–31.86)	6.29 (3.70–9.54)	>0.05^#^
*N* (%)	48.06 ± 25.25	42.74 ± 20.18	43.15 ± 14.99	>0.05^*^
HB (g/L)	119.75 ± 9.54^a^	124.76 ± 7.86^b^	125.42 ± 10.19^c^	<0.05^*^
PLT (×10^9^/L)	200.56 ± 70.95^a^	256.86 ± 111.47^b^	251.44 ± 91.16^c^	<0.05^*^
CRP (mg/L)	2.00 (0.50–90.30)	1.75 (0.50–15.80)	1.65 (0.50–7.10)	>0.05^#^
Glucose (mmol/L)	5.50 (3.48–8.61)	5.33 (4.77–8.64)	5.25 (4.59–8.64)	>0.05^#^
Sodium (mmol/L)	137 (130–142)^a^	138 (133–149)^b^	138 (134–149)^c^	<0.05^#^

*FS, febrile seizures; FC, febrile control; HC, healthy control; WBC, white blood cell; HB, hemoglobin; N, neutrophil; PLT, platelet; CRP, C-reactive protein*.

# *The P-value is for Kruskal-Wallis test*;

* *The P-value is for One-way ANOVA test*.

a, b, c *on means refer to significant difference between means when K-W or One-way ANOVA test refers to significance by multiple comparison analysis (bc, non-significant; ab, ac, significant)*.

d *Chi-square test*.

e *Data shown mean ± standard deviation and median (min–max)*.

Table 2 shows the changes in serum levels of leptin, adiponectin, interleukin-6, and visfatin in the three groups. Serum leptin and visfatin levels in the FS and FC groups were significantly lower than those in the HC group (*P* < 0.01), but there was no difference between the FS and FC groups. The levels of serum adiponectin and IL-6 in FS children were significantly higher than those in the FC and HC groups (*P* < 0.01), but there was no difference between FC and HC groups.

**Table 2:** Comparison of adipocytokines between the patient group and control groups^a^.

	**FS group (*n* = 55)**	**Fc group (*n* = 42)**	**Hc group (*n* = 48)**	***P-*value (among groups)**	***P*-value (FS and FC)**	***p*-value (FS and HC)**	***p*-value (FC and HC)**
Leptin (pg/ml)	539.75 (9.41–2030.08)	393.93 (181.51–1545.95)	1447.87 (19.23–2948.69)	<0.05^#^	0.65^##^	<0.001^##^	<0.001^##^
Visfatin (pg/ml)	2.90 (0.04–13.50)	2.62 (0.02–9.82)	7.06 (1.25–22.84)	<0.05^#^	1^##^	<0.001^##^	<0.001^##^
Adiponectin (μg/ml)	13.09 ± 4.38	8.20 ± 2.53	7.19 ± 2.39	<0.05^*^	<0.001^**^	<0.001^**^	0.149^**^
IL-6 (pg/ml)	8.96 (4.73–38.74)	5.17 (4.61–46.09)	5.11 (4.60–31.03)	<0.05^#^	<0.001^##^	<0.001^##^	0.58^##^

*FS, febrile seizures; FC, febrile control; HC, healthy control; IL-6, Interleukin 6*.

# *The P-value is for Kruskal-Wallis test*;

* *The P-value is for One-way ANOVA test*;

## *The P-value is for Nemenyi test*;

** *The P-value is for LSD test*.

a *Data shown mean ± standard deviation and median (min–max)*.

When we compared the SFS with the CFS group, we found that the platelet count of the CFS group was significantly lower than that of the SFS group. There was no significant difference between other clinical data and laboratory findings in the two groups (Table 3).

**Table 3:** Comparison of clinical and laboratory findings between SFS group and CFS group^b^.

**Characteristics**	**SFS group (*n* = 42)**	**CFS group (*n* = 13)**	***P-*value**
Age (months)	33.00 ± 18.35	43.08 ± 28.10	>0.05^#^
Sex (male/female)^a^	27/15	5/8	>0.05
BT (°C) on admission	39.31 ± 0.63	39.48 ± 0.72	>0.05^#^
**Laboratory findings**			
WBC (×10^9^/L)	7.51 ± 3.68	7.90 ± 5.11	>0.05^#^
*N* (%)	46.67 ± 25.36	52.55 ± 25.35	>0.05^#^
HB (g/L)	120.19 ± 10.37	118.31 ± 6.26	>0.05^#^
PLT (×10^9^/L)	211.67 ± 70.84	164.69 ± 60.65	<0.05^#^
CRP (g/L)	3.05 (0.50–60.30)	1.90 (0.50–90.30)	>0.05^*^
Glucose (mmol/L)	5.69 ± 1.34	5.34 ± 0.71	>0.05^#^
Sodium (mmol/L)	136.88 ± 2.69	135.77 ± 3.53	>0.05^#^
Leptin (pg/ml)	561.60 (9.41–2030.08)	274.48 (46.56–190.78)	>0.05^*^
Visfatin (pg/ml)	2.82 (0.04–13.50)	3.27 (0.56–8.65)	>0.05^*^
Adiponectin (μg/ml)	12.99 ± 4.59	13.40 ± 3.75	>0.05^#^
IL-6 (pg/ml)	9.34 (4.73–38.74)	8.50 (4.75–37.70)	>0.05^*^

*SFS, Simple febrile seizures; CFS, Complex febrile seizures; BT, body temperature; WBC, white blood cell; HB, hemoglobin; N, neutrophil; PLT, platelet; CRP, C-reactive protein; IL-6, Interleukin 6*.

# *The P-value is for independent t-test*;

* *The P-value is for Mann-Whitney U-test*.

a *Chi-square test*.

b *Data shown mean ± standard deviation and median (min–max)*.

All children with FS were followed up until November 2019, and 22 cases of recurrence were found, of which 17 relapsed in the SFS group and five relapsed in the CFS group. There were no significant differences in serum leptin, visfatin, adiponectin, or IL-6 between the relapsed and non-relapsed groups (Table 4).

**Table 4:** Comparison of adipocytokines between the recurrent group and non-recurrent group^a^.

	**Recurrent group (*n* = 22)**	**Non-recurrent group (*n* = 33)**	***P-*value**
Leptin (pg/ml)	478.44 ± 386.41	696.36 ± 454.64	0.07^#^
Visfatin (pg/ml)	2.04 (0.56–8.65)	3.06 (0.04–13.50)	0.536^*^
Adiponectin (μg/ml)	13.22 ± 4.16	13.00 ± 4.58	0.852^#^
IL-6 (pg/ml)	10.00 (4.73–37.70)	8.38 (4.75–38.74)	0.536^*^

*IL-6, Interleukin 6*.

# *The P-value is for independent t-test*;

* *The P-value is for Mann-Whitney U-test*.

a *Data shown mean ± standard deviation and median (min–max)*.

There was no correlation between leptin, adiponectin, visfatin, and IL-6 in the FS and FC groups. There was a weak negative correlation between leptin and adiponectin in the HC group (Tables 5–7). There is a slight negative correlation between serum sodium and FS (the correlation coefficient between serum sodium and FS is *r* = −0.329, *P* < 0.001). The detailed urine volume was not monitored, nor was urine sodium and creatinine detected, however, further studies are recommended. There is a positive correlation between body temperature and FS: *r* = 0.46, *P* < 0.001. In addition, no correlation was observed between seizures and age.

**Table 5:** Correlation analysis of adipokines in FS group.

**Exercise parameter**		***r***	***P*-value**
**Leptin**			
	Visfatin	0.039	0.777
	Adiponectin	−0.159	0.247
	IL-6	0.179	0.192
**Visfatin**			
	Adiponectin	−0.039	0.779
	IL-6	0.11	0.423
**Adiponectin**	IL-6	−0.123	0.37

**Table 6:** Correlation analysis of adipokines in FC group.

**Exercise parameter**		***r***	***P*-value**
**Leptin**			
	Visfatin	−0.238	0.128
	Adiponectin	0.139	0.38
	IL-6	0.224	0.153
**Visfatin**			
	Adiponectin	0.094	0.555
	IL-6	−0.259	0.098
**Adiponectin**	IL-6	0.12	0.448

**Table 7:** Correlation analysis of adipokines in HC group.

**Exercise parameter**		***r***	***P*-value**
**Leptin**			
	Visfatin	0.182	0.217
	Adiponectin	−0.385	**0.007**
	IL-6	−0.082	0.581
**Visfatin**			
	Adiponectin	0.052	0.724
	IL-6	−0.193	0.188
**Adiponectin**	IL-6	0.236	0.106

*IL-6, Interleukin 6*.

*P < 0.05 is marked in bold*.

In the linear regression analysis, high serum adiponectin and IL-6 levels were significantly associated with the risk of febrile seizures among studied FS patients, but leptin and visfatin were not associated with FS. When binary logistic regression analysis was performed, the high serum adiponectin levels were the most significant risk factor associated with FS among studied children [odds ratio (OR): 1.669; 95% confidence interval (CI): 1.388–2.007; *P* = 0.000], and high serum IL-6 levels were also significantly associated with FS (OR: 1.079; 95% CI:1.022–1.139; *P* = 0.006) (Table 8).

**Table 8:** Binary logistic regression analysis of serum adiponectin and IL-6 levels as risk factors for febrile seizures among studied subjects.

**Risk factor**	**Coefficient**	***P*-value**	**Odds ratio**	**95% CI^a^**
Adiponectin (μg/ml)	0.512	0.000	1.669	1.388–2.007
IL-6 (pg/ml)	0.076	0.006	1.079	1.022–1.139

*IL-6, Interleukin 6*.

a *CI indicates confidence interval*.

In addition, we made a comparative analysis of serum indicators of children <3 years old and older than 3 years old. The group under 3-years-old included 74 children (mean age 1.8 ± 0.7 years), of which 33 were FS patients (mean age 1.8 ± 0.7 years), 18 were FC patients (mean age 1.9 ± 0.6 years), and 23 were healthy controls (mean Age 1.7 ± 0.8 years). The results of leptin, visfatin, and adiponectin in the group under 3-years-old were the same as the results of the whole group mentioned above. Serum IL-6 levels were significantly higher in children with FS compared to the HC group (*P* < 0.01), but there was no difference between either the FS and FC groups or the FC and HC groups (Table 9).

**Table 9:** Comparison of adipocytokines between the patient group and control groups under 3^a^.

	**FS group (*n* = 33)**	**Fc group (*n* = 18)**	**Hc group (*n* = 23)**	***P-*value (among groups)**	***P*-value (FS and FC)**	***p*-value (FS and HC)**	***p*-value (FC and HC)**
Leptin (pg/ml)	539.75 (46.56–2030.08)	389.31 (181.51–1545.95)	1507.06 (101.43–2896.33)	<0.05^#^	0.96^##^	<0.001^##^	<0.001^##^
Visfatin (pg/ml)	2.68 (0.04–8.65)	2.78 (0.02–9.26)	6.84 (1.56–22.84)	<0.05^#^	1^##^	<0.001^##^	<0.001^##^
Adiponectin (μg/ml)	13.58 ± 4.77	8.50 ± 2.88	6.99 ± 2.54	<0.05^*^	<0.001^**^	<0.001^**^	0.237^**^
IL-6 (pg/ml)	8.50 (4.77–38.52)	5.50 (4.63–46.09)	5.16 (4.62–31.03)	<0.05^#^	0.06^##^	<0.001^##^	0.71^##^

*FS, febrile seizures; FC, febrile control; HC, healthy control; IL-6, Interleukin 6*.

# *The P-value is for Kruskal-Wallis test*;

* *The P-value is for One-way ANOVA test*;

ȴ# *The P-value is for Nemenyi test*;

** *The P-value is for LSD test*.

a *Data shown mean ± standard deviation and median (min–max)*.

The 3+ year group included 71 children (mean age 4.9 ± 1.3 years), including 22 patients with FS (mean age 4.7 ± 1.4 years), 24 FC patients (mean age 4.8 ± 1.3 years), and 25 healthy controls (mean age: 5.1 ± 1.4 years). The results for visfatin, adiponectin, and IL-6 were the same as those for the entire group described above. The leptin levels in the FC group were significantly lower than those in the HC group (*P* < 0.01), but there was no difference between the FS group and the control group (Table 10).

**Table 10:** Comparison of adipocytokines between the patient group and control groups over 3^a^.

	**FS group (*n* = 22)**	**FC group (*n* = 24)**	**Hc group (*n* = 25)**	***P-*value (among groups)**	***P*-value (FS and FC)**	***p*-value (FS and HC)**	***p*-value (FC and HC)**
Leptin (pg/ml)	628.01 (9.41–1381.17)	476.26 (212.30–1002.90)	1155.75 (19.23–2948.69)	<0.05^#^	1.45^##^	0.06^##^	<0.001^##^
Visfatin (pg/ml)	3.27 (0.56–13.50)	2.49 (0.92–9.82)	7.38 (1.25–22.35)	<0.05^#^	0.99^##^	0.02^##^	0.01^##^
Adiponectin (μg/ml)	12.35 ± 3.70	7.98 ± 2.27	7.36 ± 2.28	<0.05^*^	<0.001^**^	<0.001^**^	0.446^**^
IL-6 (pg/ml)	10.48 (4.73–38.74)	4.82 (4.61–29.82)	5.11 (4.60–19.31)	<0.05^#^	0.01^##^	<0.001^##^	0.82^##^

*FS, febrile seizures; FC, febrile control; HC, healthy control; IL-6, Interleukin 6*.

# *The P-value is for Kruskal-Wallis test*;

* *The P-value is for One-way ANOVA test*;

## *The P-value is for Nemenyi test*;

** *The P-value is for LSD test*.

a *Data shown mean ± standard deviation and median (min–max)*.

## Discussion

This study measured blood routine, biochemical indicators, serum leptin, adiponectin, IL-6, and visfatin levels in children with FS, as well as in children of the same age with febrile illness without seizures (FC), and healthy controls (HCs). We also evaluated the correlation with clinical indicators by logistic regression analysis. The main findings of this study were that serum adiponectin and IL-6 levels were significantly higher in the FS group than in the FC and HC groups, while there was no statistical difference between the FC and HC groups.

FS mostly occurs in children from 6 months to 6 years old, but it can sometimes occur in children of an older age. These cases have a history of FS. In this study, there were three children with FS older than 6 years of age, all of whom had a history of FS before the age of 6, including two patients who had experienced FS twice and one patient who had experienced it five times. The three children were excluded from diseases, such as intracranial infection, space occupation, epilepsy, and genetic and metabolic abnormalities, and were diagnosed as FS after blood biochemistry, electroencephalography, cranial magnetic resonance imaging (MRI), and cerebrospinal fluid examination.

Comparison of clinical and laboratory findings of patient and control groups showed that Hb, platelet counts, and blood sodium levels were significantly lower in the FS group than those in the FC and HC groups. It has been shown that iron deficiency anemia may be related to the increased risk of children suffering from FS and is one of the risk factors for FS (15). Similarly, studies have found that the reduction of peripheral blood platelet counts in children with FS may be related to the release of a large number of inflammatory mediators caused by platelet activation caused by infection (16, 17). In addition, previous studies have found that children with FS are prone to hyponatremia (18, 19), which may be the result of FS, but it remains unclear whether it can predict the onset of FS (20, 21). Whether serum Hb level, platelet count, and sodium level can help predict the prognosis of FS is worthy of further study.

Analysis of the four adipokines showed that some of the results were similar to previous studies (8, 11–13), but this study also has new findings. A study by Turkish Güven et al. (11) found that the levels of adiponectin and IL-6 in children under the age of 3 in the FS group were significantly higher than those in the HC group (*p* < 0.05), which is consistent with the results of the present study, but there was no statistical difference in serum adiponectin and IL-6 levels between the FS and FC groups in Güven's study. However, in the current study, the adiponectin level in the FS group was also significantly higher than that in the FC group, and there was no statistical difference between the FC and HC groups, while in the Güven study, the adiponectin level in the FS group was not statistically different from the FC group. The main reason for this difference may be the sample size. In Güven's study, the sample size was relatively small, with FS = 33, FC = 26, and HC = 29 for each group. In this study, the sample sizes were FS = 55, FC = 42, and HC = 48. Other factors, such as antipyretic treatment and their relationship to the measured proteins, may also influence the results, which merits further investigation. For example, in this study, children in the FS group and the FC group used ibuprofen to reduce fever, and animal experiments suggest that ibuprofen can reduce the concentration of serum leptin and IL-6 (22). In Güven's study, information about antipyretics is not provided.

In addition, it should be noted that the age of previous studies (8, 11–13) is <3-years-old. In this study, the age range is large, and there is no difference in the number of children over 3 years old and children under 3 years old. Therefore, we chose 3-years-old as the cut-off value between the groups. This not only enables better matching and comparisons with previous similar work, but also further expands previous related research. Our results from children over 3 years of age showed that many indicators were consistent with those <3 years old. For example, serum adiponectin levels in the FS group were significantly higher than those in the FC and HC groups, while there was no significant difference between the FC and HC groups. In addition, the level of IL-6 in the FS group was higher than that in the HC groups, but there was no difference in IL-6 levels between the FC and the HC groups. Logistic regression analysis further showed that high concentrations of adiponectin and IL-6 are related to the occurrence of FS. High serum adiponectin levels are the biggest risk factors for FS. High IL-6 levels are also the cause of FS risk factors, while leptin and visfatin were not related to the occurrence of FS. Therefore, it is reasonable to speculate that adiponectin and IL-6 levels can be used to predict the occurrence of FS.

Adiponectin is secreted by adipocytes and its basic function is to improve insulin sensitivity and fat oxidation (23). Later, widely expressed adiponectin receptors were found in the brain (24), and low concentrations of adiponectin were also detected in the cerebrospinal fluid (25), suggesting that adiponectin is involved in the regulation of brain metabolism and function. It is worth noting that adiponectin may have a neuroprotective effect in epilepsy. Adiponectin deficiency in mice on a high-fat diet led to increased seizure severity and hippocampal pathological changes (26). Neuronal damage caused by seizures is related to blood-brain barrier (BBB) leakage. Adiponectin retains the integrity of BBB and has neuroprotective effects in animal models of seizures caused by KA (27), suggesting that adiponectin plays an anticonvulsant role by protecting the integrity of vascular endothelial cells. Further research found that the protective effect of adiponectin on vascular endothelial cells may be related to its anti-inflammatory effect. Adiponectin can significantly inhibit the production of tumor necrosis factor-α (TNF-α) in macrophages (28). Adiponectin is protective against ischemic brain injury by modulating inflammatory pathways and endothelial function, and a low level of plasma adiponectin is associated with increased mortality after ischemic stroke (29, 30). Adiponectin affects the risk of dementia and its pathophysiology through its anti-inflammatory and anti-atherosclerotic effects (31). Notably, both adiponectin and IL-6 are closely related to epilepsy. Previous studies have found that in patients with refractory epilepsy, serum adiponectin levels are lower than healthy controls, and serum IL-6 levels are higher (32). A recent meta-analysis showed an association between IL-6 (572,174,597) polymorphisms and susceptibility to FS. T alleles and TT genotypes may be associated with an increased risk of FS (33). Therefore, elevated serum adiponectin levels in children with FS may play an anti-inflammatory role and reduce IL-6-mediated inflammatory responses, thereby reducing brain damage caused by FS. This potential mechanism of neuroprotection may be related to the regulation of the BBB integrity.

There is limited information about the correlation between levels of leptin and visfatin with FS. Leptin is mainly secreted by white adipose tissue and regulates energy homeostasis by inhibiting food intake and reducing weight (34). In addition to its role in mammalian metabolism, circulating leptin can cross the BBB and act as a neurotrophic factor through its receptors, thereby regulating neural plasticity and cognitive function (35–37). Moreover, leptin has been shown to regulate both innate and adaptive immune responses in both normal and pathological conditions (38, 39). Leptin is an acute phase reactant and is considered as an inflammatory mediator, which contributes to the thermal changes in systemic inflammation (40). Previous studies have reported both anti-seizure and pro-seizure properties of leptin (41–44). Visfatin (nicotinamide phosphoribosyltransferase, NAMPT) is an enzyme which catalyzes the biosynthesis of nicotinamide adenine dinucleotide (NAD+) in mammals. Adipose-secreted visfatin serves as a neuroendocrine factor and has a strong impact on modulating brain functions (45). Visfatin protects neurons against ischemia-induced injury (46).

The earliest article on the relationship between FS and leptin published by Korean scholars reported that there was no difference in serum leptin levels between the FS group and the normal control. Güven et al. (11) later found that the leptin levels in the FS and FC groups were higher than those in the normal control group, while the study by Seham (12) showed that the leptin levels in the FS group were lower than those in the normal control group, but the leptin levels in the FC group were significantly higher than in the HC group. Here, we found that the leptin and visfatin levels in the FS and FC groups were significantly lower than those in the normal control group, but there was no statistical difference between the FS and FC groups. To the best of our knowledge, this is the first study to measure serum visfatin levels in children with FS. The reasons for these inconsistencies may be related to differences in sampling age, blood collection time, and primary diseases that cause fever. A multicenter follow-up study of the role of leptin and visfatin in children with FS and fever is recommended.

In addition, no significant difference was found between the SFS group and the CFS group, whether it was leptin, visfatin, or adiponectin and IL-6. There was also no difference between the relapsed and non-relapsed groups. Therefore, these factors cannot be used to predict CFS.

This study has some limitations. The blood samples were taken after the FS, meaning the FS itself might have induced the observed changes (in which case the diagnostic role of the proteins is negligible). It should be noted, however, that it is currently clinically impossible to accurately determine the exact time of a seizure in order to collect a blood sample before the seizure begins. Therefore, it is currently common practice to collect blood samples immediately after a seizure. For example, in an article published in 2020 by Costea et al. (47), the authors analyzed the predictive value of plasma biochemical marker molecules within half an hour after FS, which has a certain reference value for assisting the prognosis of seizure recurrence in children with FS.

In conclusion, this study suggests that higher plasma levels of IL-6 and adiponectin could serve as an additional biomarker in the early treatment or follow-up of FS children, which merits further investigation in large-scale multicenter studies.

## Data Availability Statement

All datasets generated for this study are included in the article/supplementary material.

## Ethics Statement

The studies involving human participants were reviewed and approved by the Human Ethics Committee of the Wuxi Second People' Hospital China. Written informed consent to participate in this study was provided by the participants' legal guardian/next of kin.

## Author Contributions

HN was the designer and dissertation writer of this study. JC, MJ, LT, and YL were the operators of this experiment and were responsible for the statistical analysis of the data. All authors contributed to the article and approved the submitted version.

## Conflict of Interest

The authors declare that the research was conducted in the absence of any commercial or financial relationships that could be construed as a potential conflict of interest.
